# A novel custom high density-comparative genomic hybridization array detects common rearrangements as well as deep intronic mutations in dystrophinopathies

**DOI:** 10.1186/1471-2164-9-572

**Published:** 2008-11-28

**Authors:** Matteo Bovolenta, Marcella Neri, Sergio Fini, Marina Fabris, Cecilia Trabanelli, Anna Venturoli, Elena Martoni, Elena Bassi, Pietro Spitali, Simona Brioschi, Maria S Falzarano, Paola Rimessi, Roberto Ciccone, Emma Ashton, Joanne McCauley, Shu Yau, Stephen Abbs, Francesco Muntoni, Luciano Merlini, Francesca Gualandi, Alessandra Ferlini

**Affiliations:** 1 Sezione di Genetica Medica, University of Ferrara, Italy; 2 U.O. Genetica Medica St. Anna Hospital, Italy; 3 Sezione di Biologia generale e Genetica Medica, University of Pavia, Italy; 4 DNA Laboratory, Genetics Centre, Guy's & St. Thomas NHS Foundation Trust, London, UK; 5 UCL Institute of Child Health, Dubowitz Neuromuscular Centre, London, UK

## Abstract

**Background:**

The commonest pathogenic *DMD *changes are intragenic deletions/duplications which make up to 78% of all cases and point mutations (roughly 20%) detectable through direct sequencing. The remaining mutations (about 2%) are thought to be pure intronic rearrangements/mutations or 5'-3' UTR changes. In order to screen the huge *DMD *gene for all types of copy number variation mutations we designed a novel custom high density comparative genomic hybridisation array which contains the full genomic region of the *DMD *gene and spans from 100 kb upstream to 100 kb downstream of the 2.2 Mb *DMD *gene.

**Results:**

We studied 12 DMD/BMD patients who either had no detectable mutations or carried previously identified quantitative pathogenic changes in the *DMD *gene. We validated the array on patients with previously known mutations as well as unaffected controls, we identified three novel pure intronic rearrangements and we defined all the mutation breakpoints both in the introns and in the 3' UTR region. We also detected a novel polymorphic intron 2 deletion/duplication variation. Despite the high resolution of this approach, RNA studies were required to confirm the functional significance of the intronic mutations identified by CGH. In addition, RNA analysis identified three intronic pathogenic variations affecting splicing which had not been detected by the CGH analysis.

**Conclusion:**

This novel technology represents an effective high throughput tool to identify both common and rarer DMD rearrangements. RNA studies are required in order to validate the significance of the CGH array findings. The combination of these tools will fully cover the identification of causative DMD rearrangements in both coding and non-coding regions, particularly in patients in whom standard although extensive techniques are unable to detect a mutation.

## Background

The *DMD *gene was the first gene identified by reverse genetics. Mutations in the gene cause Duchenne (DMD) and Becker (BMD) muscular dystrophies. Both the frequency and devastating nature of these conditions make *DMD *one of the most extensively studied genes among the rare genetic disorders [1-3].

This intense research has provided molecular tools for the identification of the causative mutation in about 98% of patients, combining MLPA to detect exonic deletions/duplications (75–80% of mutations) and direct sequencing to identify small mutations (up to 20% of mutations). Nevertheless, some mutations remain unidentified. Furthermore it is well known that the large size (2.2 Mb) of the gene makes it prone to complex rearrangements which are impossible to define precisely using routine molecular diagnostic techniques.

As a consequence, there are a considerable number of DMD/BMD patients in whom no causative mutation has been identified. This impacts on genetic diagnosis, genetic prognosis, clinical confirmation, carrier detection, prenatal diagnosis and genetic counselling for the families involved.

Furthermore, the recent opportunities in terms of innovative therapeutic approaches [4,5] highlight the relevance for patients and families of obtaining a correct molecular diagnosis, which is required in order to be included in innovative trials. Indeed the increased availability of experimental but highly mutation specific therapies, summarised in the concept of "personalised medicine" [6,7], makes the identification of private mutations in the *DMD *gene necessary to be eligible for these trials.

In the last few years genome scanning technologies have enabled the detection of previously unrecognised large (>1 kb) copy-number variations (CNVs) in human DNA. While many of these variants do exist as polymorphisms, some of them can change the copy number of critical genes or genomic regions, or alter gene regulation and underlie monogenic disorders, developmental abnormalities and a variety of complex genetic disorders [8-11].

Therefore there is a wide consensus on the potential of array-CGH to determine CNVs for research and clinical purposes, in terms of providing robust and precise measurement of CNVs, scalability and very high resolution [12].

Although CGH was initially considered as a strategy for improving cytogenetic resolution by detecting fine chromosome imbalances [13,14], recently other applications have been envisaged such as cancer studies [15], complex syndromes, mental retardation, Mendelian disorders and polygenic traits [16].

The flexibility of CGH arrays is also due to the availability of both commercial and custom arrays, which are designed on demand, therefore it is possible to investigate any region of interest with the appropriate resolution.

Dhami et al. [2] designed a single strand PCR-based CGH array in order to detect exon deletions/duplications in a few genes, including *DMD*.

This strategy demonstrated the ability to identify CNVs, however, in the same way as MLPA and other techniques, it only investigated coding regions.

We have applied the CGH technique in a novel full-gene approach which investigates the presence of CNVs in the entire genomic region of the *DMD *gene. Our custom designed high density-comparative genomic hybridisation array (DMD-CGH) based on in situ synthesis of 60 mer probes with intervals of 260 bp, allowed us to obtain a full map of CNVs in the gene, including the non coding regions which have not been investigated previously.

Our studies allowed us to validate our array for accurately detecting previously identified rearrangements, to define intronic breakpoints precisely and to identify three pathogenic purely intronic CNVs. We corroborated the CGH studies by RNA analysis, therefore validating the significance of the gene imbalances identified. Transcription analysis of the full *DMD *transcript furthermore disclosed three rare splicing mutations due to small intronic changes, missed by the CGH analysis.

## Results

### DMD-CGH array analysis

**Figure 1:**
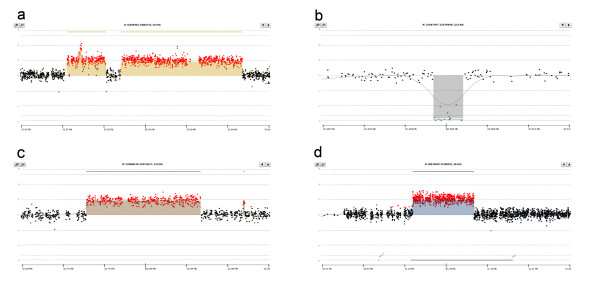
**DMD-CGH array profiles of deletions and duplications in patients with known mutations, identified by MLPA**. a) case 1, duplication of exon 2; b) case 2, deletion of exon 14; c) case 3, duplication of exons 3–6; d) case 4, duplication of exons 65–79. In figure 1a and c the intron 2 CNV is visible around 32.9 Mb.

We firstly validated the DMD-CGH array both on ten normal control males and on four patients (1, 2, 3 and 4) with mutations previously characterised by MLPA (Figure 1a–d). In patient 1, we detected two non-contiguous duplications, one of 116 kb from intron 1P to intron 2 and including exon 2 and the other of 37 kb in intron 2 (Figure 1a). Patient 2 showed a deletion of 3569 bp, from intron 13 to exon 14 (Figure 1b).

We also precisely defined the breakpoint in BMD patient number 3, carrying an out of frame exon 3–6 duplication and representing an exception to the rule [17]. The DMD CGH array identified a duplication of 111 kb from intron 2 to nt 45 of exon 6, removing its 5'donor splice site consensus sequences (Figure 1c).

In patient 4, with a duplication of exons 65–79 and a DMD phenotype with associated severe mental retardation, the array allowed us to define the 3' breakpoint within the 3' UTR. The mutation consists of a duplication of 89 kb ranging from intron 64 to 241 bp downstream of the *DMD *stop codon within the 3' UTR (Figure 1d).

The DMD-CGH allowed us to identify the causative rearrangements in three out of eight DMD patients previously negative for *DMD *mutations.

**Figure 2:**
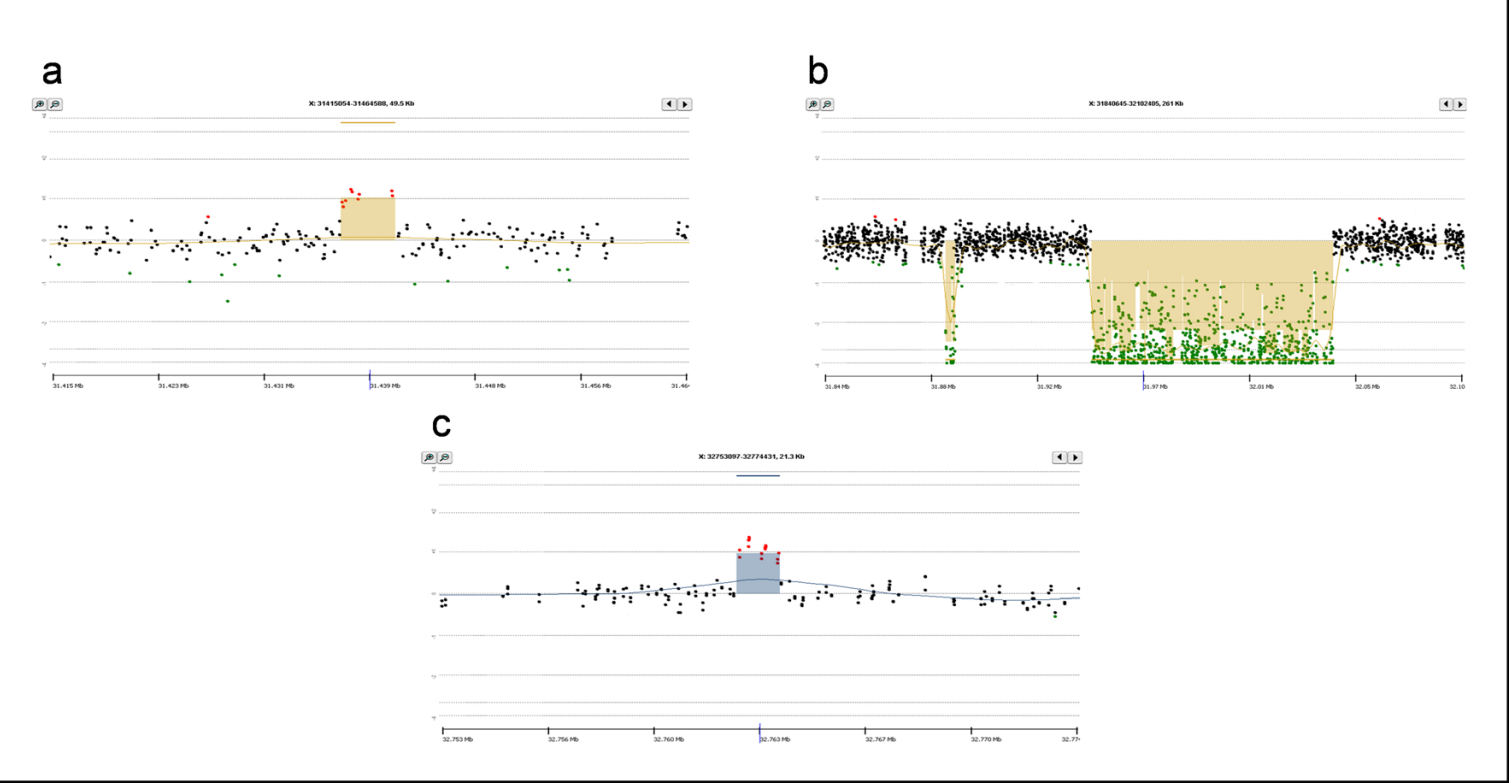
**DMD-CGH array profiles of deletions and duplications in patients negative for *DMD *mutations**. a) case 5; b) case 6; c) case 7.

We identified a 4 kb duplication in intron 55 located 12 kb upstream of exon 56 (patient 5, Figure 2a); two non-contiguous deletions of 98 kb in intron 44 and 4 kb in intron 45 (patient 6, Figure 2b); and a duplication of 1.3 kb in intron 4 located 11 kb from exon 5 (patient 7, Figure 2c). In the remaining five patients (8; 9; 10; 11; 12) no pathogenic CNVs were identified by CGH.

**Figure 3:**
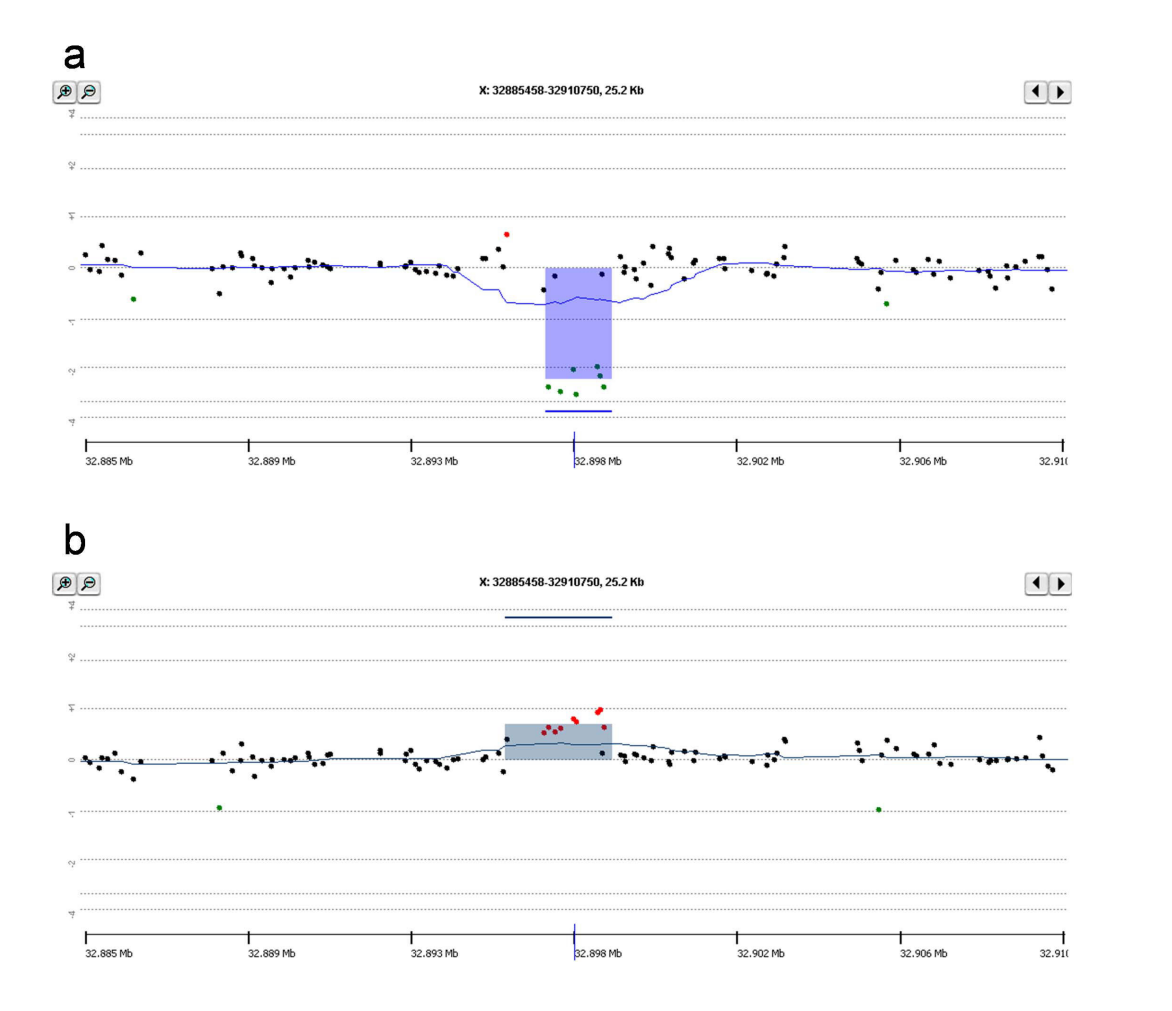
**DMD-CGH array profiles of intron 2 CNV**. a) deleted allele and b) duplicated allele.

Interestingly in all 12 patients the DMD-CGH array identified a CNV of 1.4 kb in intron 2 which was deleted in patients 10 and 11 and duplicated in all the others (Figure 1a and 1c). Examples of deleted and duplicated alleles of the intron 2 CNV are reported in Figure 3.

CGH analysis of ten normal control males revealed the presence of both deleted and duplicated regions, therefore suggesting this to be a polymorphic CNV (data not shown).

Real Time PCR was performed in patients 5 and 7 while PCR and sequencing were performed in patients 6, 10 and 11, validating the duplications and deletions identified with the array (data not shown).

### RNA analysis and sequence analysis

#### Patients with pathogenic CNVs identified at the DMD-CGH array analysis

Patient 5 showed a 4 Kb duplication in intron 55 confirmed by RealTime PCR, but no RNA was available for analysis to determine whether this variant was pathogenic or not.

**Figure 4:**
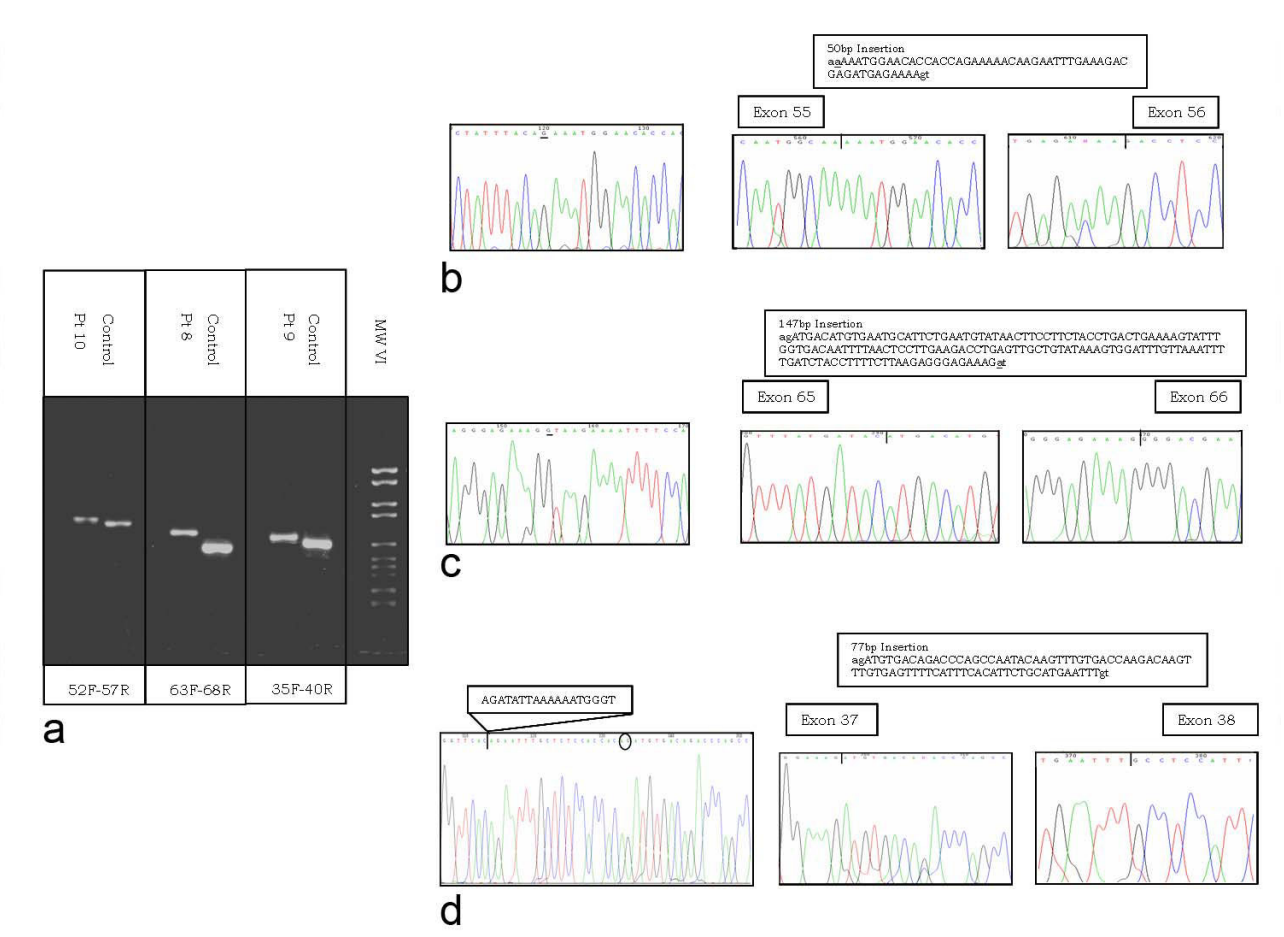
**DNA and RNA analysis in patients 10, 8 and 9**. a) RT-PCR analysis: the amplification of exons 52–57, 63–68, 35–40 respectively, resulted in fragments larger than controls. b) *Right*: direct sequencing of the exons 52–57 amplification product in patient 10 showed an insertion of 50 bp (upper box) between exons 55 and 56, derived from intron 55; *Left*: sequence of the intron 55 specific region showed an A to G transition that lead to the creation of an acceptor splice site; c) *Right*: direct sequencing of the exons 63–68 amplification product in patient 8 showed an insertion of 147 bp (upper box) derived from intron 65 between exons 65 an 66; *Left*: sequence of the intron 65 specific region showed an A to G transition in intron 65 that result in the creation of a donor splice site; d) *Right*: direct sequencing of the exons 35–40 amplification product in patient 9 showed an insertion of 77 bp (upper box) derived from intron 37 between exons 37 and 38; *Left*: the genomic sequence surrounding the intron 37 region inserted into the mature transcript revealed the presence of a canonical acceptor and donor splice sites flanking the sequence itself. Moreover we identified a deletion of 18 nucleotides (reported in the box) which is located 20 nucleotides upstream in respect to the sequence inserted into the transcript.

DMD-CGH analysis of patient 6 revealed two non-contiguous deletions located in intron 44 and intron 45. RNA analysis showed skipping of exon 45 (data not shown). Since no splicing defect was detected on genomic DNA using FM-CSCE analysis this suggested that an inversion could be responsible for the phenotype. Based on DMD-CGH results PCR analysis was performed confirming the occurrence of an inversion of the entire region with a deletion of 98 kb (intron 44) and 4 kb (intron 45) at the respective inversion breakpoints (Figure 4a and 4b).

Patient 7 showed a 1.3 kb duplication within intron 4. The duplication was confirmed by RealTime PCR. RNA analysis showed failure to amplify the exon 4–5 junction, whereas exons 1 to 4 and exons 5 to 8 were correctly spliced. PCR analysis with primers located within the duplicated region coupled with primers in exons 4 and 5, failed to detect any product. This behaviour suggests the insertion of a very large intronic region into the transcript between exons 4 and 5.

Patient 3 with BMD and the out-of-frame MLPA exons 3–6 genomic duplication showed an in-frame exons 3–5 transcript, as expected considering the CGH results [17].

#### Patients negative for pathogenic CNVs at the DMD-CGH Array analysis

**Figure 5:**
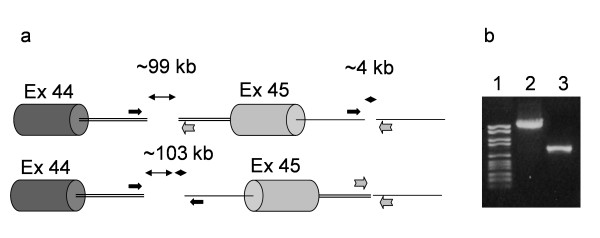
**Genomic configuration in patient 6**. a) Schemes of the inverted genomic region including exon 45 in patient 6 and primers position. PCR amplification for the detection of inversion breakpoints was carried out using two forward primers (black arrows) and two reverse primers (grey arrows) surrounding the breakpoint regions; b) PCR results in patient 6: Lane 1 molecular weight marker VI, Lane 2: proximal breakpoint (int44/int45(inv), Lane 3: distal breakpoint (int44(inv)/int45).

Patient 10 showed an insertion of 50 bp between exons 55 and 56, derived from intron 55 (Figure 5a). In this patient the sequence of the intron 55 specific region showed an A to G transition that lead to the creation of an acceptor splice site (Figure 5b left). The recognition of a donor splice site 50 nucleotides downstream results in the incorporation of the intronic sequence into the mature transcript, causing a frameshift. (Figure 5b right).

Patient 8 showed an insertion of 147 bp derived from intron 65 between exons 65 and 66 (Figure 5a). In this patient an A to G transition in intron 65 lead to the creation of a donor splice site (Figure 5c left). The presence of an acceptor splice site 147 nucleotides upstream caused the incorporation of the intronic region into the mature transcript (Figure 5c right).

Patient 9 showed an insertion of 77 bp derived from intron 37 between exons 37 and 38 (Figure 5a). This sequence is normally present within intron 37 and flanked by two cryptic splice sites. However a deletion of 18 nucleotides immediately upstream of the cryptic acceptor splice site also occurred in this patient, inducing the recognition and incorporation of the abnormal out-of-frame exon into the transcript (Figure 5d, left and right).

### Intron 2 CNV

RNA analysis in both patients 8 (with the 1.4 duplication in the same region) and 10 (with the 1.4 Kb deletion in intron 2) showed a correct amplification of the transcript including exon 2 and 3, therefore excluding a splicing abnormality as expected since the same CNV was also found in the 10 unaffected males (data not shown).

All the results of the CGH study are reported in Table 1.

**Table 1:** Summary of clinical, genomic and RNA data for analysed patients

**Sample**	**Clinical phenotype**	**MLPA results**	**Array results**	**Array breakpoints**	**Size of breakpoint**	**RNA results**
**Controls**						
Pt 1	DMD	dup ex2	dup (intron 1P_intron 2)+ dup intron 2	chrX:g.(32,882,565_32,885,186)_(32,922,352_32,923,179)dup+ (32,936,752_32,938,681)_(33,055,162_33,055,546)dup	37,166 + 116,481	r.(ex2)dup
Pt 2	BMD	del ex14	del (intron 13_ex14)	chrX:g.(32,501,829_32,502,002)_(32,505,571_32,505,939)del	3,569	Not available
Pt 3	BMD	dup ex3_6	dup (intron 2_ex6)	chrX:g.(32,744,336_32,744,610)_(32,855,933_32,856,897)dup	111,323	r.(ex3_5)dup
Pt 4	DMD plus MR	dup ex65_79	dup (intron 64_*241)	chrX:g.(31,049,450_31,049,716)_(31,138,870_31,139,129)dup	89,154	Not available
**Patients**						
Pt 5	DMD	NEG	dup intron 55	chrX:g.(31,437,578_31,437,692)_(31,441,662_31,442,049)dup	3,970	Not available
Pt 6	DMD	NEG	del intron 44 + del intron 45	chrX:g.(31,890,926_31,891,072)_(31,894,874_31,894,891)del+ (31,950,161_31,950,611)_(32,048,920_32,049,217)del	3,802 + 98,309	r.(ex45)del
Pt 7	DMD	NEG	dup intron 4	chrX:g.(32,762,962_32,763,096)_(32,764,472_32,764,484)dup	1,376	r.0(ex4_5)
Pt 8	DMD	NEG	NEG	-	-	r.9563+1215A>G ins9563+1068_9563+9563+1214
Pt 9	DMD	NEG	NEG	-	-	r.(5325+1740_5325+1757)del ins5325+1779_5325+1839
Pt 10	DMD	NEG	NEG	-	-	r.8217+18052A>G ins8217+18053_8217+18102
Pt 11	DMD	NEG	NEG	-	-	-
Pt 12	DMD	NEG	NEG	-	-	-

DMD: Duchenne Muscular Dystrophy

BMD: Becker Muscular Dystrophy

MR: Mental Retardation

NEG: Negative

Pt: Patient

## Discussion

The array CGH technique represents an extremely effective tool for the identification of CNVs in the genome with important technical advantages (especially the large scale/high resolution capacity) and relevant diagnostic implications. CGH array has been well validated in a variety of approaches for defining both cytogenetic abnormalities and some Mendelian disorders (CGH exon arrays). However, in the latter, non coding regions were never explored [2].

Here we describe the results obtained using a novel custom DMD-CGH array covering the full genomic region of the *DMD *gene, including 100 kb upstream and downstream of the 5' and 3' UTRs. We made this novel microarray in order to identify all possible quantitative pathogenic changes in the *DMD *gene as well as elusive deep intronic pathogenic CNVs. The DMD-array was able to accurately identify and refine already known deletions/duplications in the gene. This suggests that the array could be used as a high throughput technique for high scale *DMD *molecular diagnosis. Remarkably it allowed us to define both intronic and untranslated region (UTR) breakpoints in all patients studied. It also revealed rare pure intronic mutations which were not detected by routine genomic analysis. Notably, we describe a rare *DMD *gene inversion affecting exon 45 [18], the first to be identified by CGH.

As expected, the array failed to identify very small intronic mutations affecting splicing, for which RNA profiling was necessary.

Among the patients studied, we identified three novel pathogenic CNVs, which are purely intronic. RNA analysis allowed us to demonstrate, at least for two of them, that they affect the correct splicing of the *DMD *gene. The CGH-mediated identification of these rearrangements avoided an extensive RNA analysis, often impaired by low/poor quality of the RNA obtainable from patients' tissues, in particular when only MyoD transformed myogenic cells are available.

By our DMD-CGH we also identified a non pathogenic CNV within intron 2, not reported in the CNVs database [19]. This was confirmed as a normal variant by transcription analysis and by analysing normal controls.

Furthermore three complex rearrangements have been defined in term of both orientation and breakpoint definition, again improving the molecular diagnosis. This for example allowed better understanding of the genotype-phenotype correlation in a BMD patient carrying an exons 3–6 out-of-frame duplication. The DMD-CGH array showed that the 3' breakpoint falls within exon 6, providing a genomic basis for the observed splicing behaviour.

The DMD-CGH array may also help to investigate DMD/BMD cases with additional features such as severe mental retardation. Cognitive impairment in some DMD patients has been associated with mutations affecting the distal Dp140 and Dp71 dystrophin isoforms [20-22]. In our patient with the rare duplication of exons 65–79 and mental retardation, we confirmed the role of this distal region in impairing dystrophin-related brain function [20,23].

Furthermore the DMD-CGH array allowed us to reveal that the breakpoint of the large duplication within the 3' UTR involves a region containing seven AUF1 and two Hu protein binding motifs. These proteins are well known to be involved in mRNA stability [24]. It is conceivable that large genomic changes within the 3'UTR of the *DMD *gene may influence the resulting phenotype suggesting that the 3'UTR should be routinely investigated to possibly unravel still unknown *DMD *regulatory mechanisms [21,25].

Considering these results, our DMD array promises to be a useful tool both for *DMD *pathogenic CNV identification and for refining the genomic configuration not only in patients with unusual mutations but indeed in all patients. In fact, while routine mutation analysis clearly identifies apparently identical deletions in different patients, in reality the intronic breakpoints will almost invariably differ. This might involve motifs that affect gene splicing in different ways [26].

Although the advantages of using the DMD-CGH array to identify mutations are clear, RNA studies provided additional important information in these patients. In particular RNA studies allowed us to determine the significance of the CNVs identified and also to see the effects on splicing of mutations identified by the array. In addition the RNA analysis allowed to identify small mutations affecting splicing which had not been detected by the array.

Among these, we found three very unusual small deep intronic mutations which would have required extensive intronic sequencing to locate using standard methods. All three were shown to alter the *DMD *splicing profile. In particular, while the two point mutations creating a novel cryptic splice site may be considered to be easily interpreted in terms of their effect on splicing, the small 18 bp deletion within intron 37 is quite peculiar. In fact, although the effect of this deletion on the transcript is evident, it is unclear how this novel genomic configuration modifies the splicing machinery.

## Conclusion

Our results suggest that this DMD-CGH array is a valuable, cost-effective tool for high throughput *DMD *molecular diagnosis as well as for definition of elusive *DMD *gene mutations. We suggest that the CGH genomic analysis should precede RNA analysis in order to firstly define the genomic profile.

In addition to the diagnostic implications, the investigation of non-coding regions as possibly implicated in the etiopathogenesis of mutations in *DMD *but also in other genetic disorders, may disclose findings of interest for basic as well as applied research. Finally, the breakpoint definition in large rearrangements, which has always represented an extremely complex task, will considerably improve our understanding of the correlations between genotype and phenotype.

## Methods

### Patients

We have studied 12 patients with DMD/BMD by the DMD-CGH microarray, after obtaining informed consent (Table 1). Four patients were already known to have rearrangements in the *DMD *gene identified by MLPA. One DMD patient presented with mental retardation associated with a large duplication of exons 65–79. One BMD patient had an out-of-frame duplication of exons 3–6 as an exception to the reading frame rule, one DMD patient showed an isolated duplication of exon 2, and one BMD patient had a deletion of exon 14.

Eight DMD patients, fully analysed by MLPA and either sequencing or FM-CSCE [27], had tested negative for *DMD *mutations, despite protein studies using immunohistochemical and/or Western blot analysis indicating a dystrophinopathy.

Ten normal control males were also tested on the DMD-CGH array.

The study was approved by the local ethics committee (approval number 9/2005).

### Microarray design, hybridization and data analysis

DMD-CGH microarray design was performed using the web based Agilent eArray database version 4.5 [28] (Agilent Technologies, Santa Clara, CA). The high density aCGH search function within eArray was used to turn the genomic region chrX: 30947266–33367647 (March 2006 human reference sequence, NCBI Build 36.1, hg18) into a probe set by selecting the maximum number of exonic and intronic 60mer oligonucleotide CGH probes available in the database. This probe set included 9293 probes that were replicated twice in order to have both a technical replicate and to reach the array format of 4 × 44 K, creating four identical 44 K arrays on a single slide for simultaneous analysis of four different samples. 16 probes covering the exons not included in the Agilent database were designed specifically with eArray (v4.5) on the first exon of Dp71 isoform and exons 49, 50, 61 and 78 and replicated twice, achieving a final mean resolution of 260 bp. The remaining spots on each 44 K array were filled with probes from the X chromosome (11745) and all of the autosomes (12053).

Labelling and hybridisation were performed following the protocols provided by Agilent (Agilent Oligonucleotide Array-Based CGH for Genomic DNA Analysis protocol v5.0). The array was analysed with the Agilent scanner and the Feature Extraction software (v9.1). A graphical overview and analysis of the data were obtained using the CGH analytics software (v3.5). For identifying duplications and deletions we used the standard set-up of the ADM-2 statistical analysis provided by CGH analytics software. According to this set-up and in the case of X-linked genes in males, deletions are visualised with values of minus infinite (-4 in CGH analytics). The corresponding value for duplications is +1. In general, al least 3 consecutive probes reaching these values are needed for a positive call.

### Real Time PCR

We confirmed the CNVs identified by the DMD-CGH by Real time PCR analysis. Primer design on intronic regions 4 and 55 was performed by the Primer 3.0 on-line tool [29] and checked for dimer formation with OLIGO 4.0-s. Real-Time PCR was performed with Power SYBR^® ^Green PCR Master Mix (Applied Biosystems) according to the protocol supplied (User Bullettin #2). The target sequence was quantified with respect to an autosomal reference sequence (GAPDH) and to a *DMD *exon known not to be duplicated in the patient. In each experiment, control female and male DNAs were used as calibrators.

### RNA analysis

Transcription analysis of the *DMD *messenger RNA was performed in six DMD and one BMD patients.

Total RNA was isolated from muscle biopsies using the RNeasy Kit (Qiagen) following the manufacturer's instructions. In patient 9 total RNA was isolated from MyoD transformed fibroblasts as previously described [30]. Before cDNA synthesis, RNA was treated with DNAse I (Roche) and checked for residual DNA contamination with a 55 cycle PCR.

Reverse transcription (RT) and PCR amplification were performed using random hexanucleotide primers and Superscript III enzyme (Invitrogen) according to the protocol supplied. All the PCR fragments were purified using the QIAquick purification kit (QIAGEN) and sequenced on an ABI Prism 3130.

In patients 3, 6 and 7 the transcription analysis was focused on exons flanking the pathogenic rearrangement detected by DMD-CGH (exons 2–7 for patient 3, exons 43–47 for patient 6, exons 3–6 for patient 7).

In patients 8, 9 and 10 the entire *DMD *transcript was amplified in overlapping fragments of 750–800 bp (primers sequences are available upon request). For patients 5, 11 and 12 no muscle biopsies were available.

### PCR genomic analysis

In patients 8, 9 and 10 we amplified the intronic regions surrounding the sequences included in the mature transcript (introns 55, 65 and 37). DNA was extracted from leukocytes by the Qiagen Biorobot.

In Patient 6 two forward primers were coupled in order to amplify the centromeric inversion breakpoint and two reverse primers were coupled in order to amplify the telomeric inversion breakpoint.

PCR assays were performed with Ex Taq polymerase (Takara), according to standard procedures. All the PCR fragments were purified using the QIAquick purification kit (QIAGEN) and sequenced on an ABI Prism 3130. All the oligonucleotide sequences are available upon request.

## Authors' contributions

MB carried out the design of the dystrophin CGH array, hybridisation of samples, Real time PCR experiments, analysis and interpretation of data, preparation of the manuscript; MN performed RNA studies and the preparation of the manuscript, SF performed DNAs extraction and MLPA analysis, MF carried out MYOD transformation of patients fibroblasts, CT carried out *DMD *sequence analysis, AV carried out *DMD *sequence analysis, EM performed RNA extraction from patients biopsies, EB carried out the extraction of DNA samples, SP performed PCR analysis, SB carried out the collection of patients and acquisition of clinical data, MSF carried out cell cultures, PR participated in the interpretation of data and revision of the manuscript, RC supported the setting up of CGH hybridisation experiments, EA carried out the collection of patients samples, JMcC performed PCR studies and RNA studies, SY performed PCR studies and RNA analysis, SA carried out the collection of samples, FM carried out the clinical evaluation of patients, LM carried out the clinical evaluation of patients and participated in the revision of the manuscript, FG contributed to the conception of the work and preparation of the manuscript, AF carried out the conception and design of the work, the revision of the manuscript and final approval of the version.
